# Contribution to the Study of Inherited Syphilis

**Published:** 1881-12

**Authors:** 


### CONTRIBUTION TO THE STUDY OF INHERITED SYPHILIS.

Dr. H. N. Read, in a paper before the Medical Society of the

County of Kings, gave some very valuable and interesting statistics upon this important subject. Sixty-four cases are reported which are thoroughly analysed as to etiology, symptoms, prognosis and treatment. Dr. Read says: In the etiology of congenital syphilis, all minor questions as to whether a child inherits syphilis from its father or from its mother, or from both equally, or in different degrees, have gradually merged themselves into one chief question, which indeed contains all the rest, and which, if determined, settles all the others. It is: Is not a woman who has a syphilitic child necessarily in some degree syphilitic? I have no hesitation in announcing my belief in the affirmative of this question. Long hinted at, then imperfectly announced, then fully announced, then partially proved, then more fully substantiated, it now lacks but a few more links to make the chain of evidence complete, in the support of this view.

Diday evidently held his view in his work, though unable to prove it.

Mr. Hutchinson, before the Pathological Society of London, if he did not hold this view, went near to prove it (*vide* London Path. Soc. Trans., 1876), and the latest American author on this subject—Prof. Keyes of New York—whose work gives a complete resume of the subject of inherited syphilis, gives his adhesion to the doctrine, though somewhat guardedly.

It may seem a strong statement to make, that every woman who bears a syphilitic child is herself infected, in view of the numerous cases which come under the observation of almost every practitioner, of apparently healthy women having syphilitic children; but I do not mean to state that every mother of a diseased child has a chancre, or even secondary or tertiary symptoms, in the common acceptation of the terms.

The statement I do make is that every mother of a syphilitic infant is affected with a variety of the disease, which may or may not be accompanied by secondary or tertiary lesions, but which is *protective* against another attack. Mr. Hutchinson recognizes this fact in the distinction which he makes between "blood syphilis" and "chancre syphilis."

Ricord explains it on the so-called "choc en retour principle," *i. e.* the poisoning of the mother through the diseased fœtus. The proof of this doctrine lies in two points, neither of which has ever, as yet, been controverted. The first is the well-known law of Colles: That a child with inherited syphilis will never infect its own mother, though it can infect a healthy wet nurse. Since this law was first laid down by the acute observer whose name it bears, but two cases have ever been brought forward as exceptions to it. One by Brizio Cochi, an Italian, in 1858, and one by Muller, in 1861. But Kassowitz, who quotes these cases in his work, adds that they are of no value scientifically, as they are neither accurately nor distinctly described.

Since this law was enunciated, therefore, forty-three years ago, no authentic exception to it has ever been recorded among the immense number of cases observed. It may therefore stand as proved. Now if it can be shown that the mother of a syphilitic child can not only not be infected from her offspring after birth, but *from no other source*, the case stands proved.

To fill this last point of proof is exactly what the second of my two points aims at. The second point therefore is, that no case has ever been reported yet of a woman bearing a syphilitic child, who was ever *afterwards* infected with syphilis *from any source.*

This statement, of course, does not stand on a level with "Colles' law,' and will require years of observation for proof. No exception to it as yet, however, has ever been noted. Caspary attempted the only positive solution of this question. He found a seemingly healthy woman with a syphilitic husband and a syphilitic child. *He inoculated the woman with the secretion of syphilis without effect.* This is a very strong case. I desire to place the following case of my own on record as proof on this point:

"E. H., a healthy nurse-maid, sixteen years old, became pregnant by her employer, a gentleman who had contracted syphilis several years previous to this event. She continued well and strong during her pregnancy, and was delivered at full term of a fine looking child, which, however, at five weeks showed roseola mucous patches and snuffles. It was treated and recovered, had two relapses, from which it also recovered under anti- syphilitic treatment. The mother showed no signs at any time of syphilis, at least none that could be detected.

"Nearly three years after the birth of her child, she was married to a young man, whom she brought to me for treatment about three weeks after her marriage. I discovered a hard chancre on the frænum of his penis, which he assured me appeared only ten days before his marriage, and of the nature of which he was unaware.

"He afterwards acknowledged to have had impure connection with a woman of the town some weeks before the appearance of the chancre.

"He had had repeated connection with his wife, but a careful examination of her showed neither chancre, mucous patch, nor anything abnormal. The husband afterwards had secondary trouble severely, but the wife, though she has twice aborted, has never shown any symptoms of specific disease, now some sixteen or eighteen months since her marriage. This case may, I think, be regarded as strong evidence.

"The woman was repeatedly exposed to the virus of the chancre, with perfect impunity. Neither before nor since marriage has she ever shown a symptom of syphilis, though always under observation and repeatedly examined."

An analysis of sixty-four cases arranged with references to the kind of lesion developed, gives fifty-four cases in which the skin was involved. Roseola appeared among the skin diseases in thir ty-nine of the fifty-four cases, either alone or combined with some other form. Eczema was noticed in twenty cases, generally in com bination with erythema or roseola. Simple maculæ appeared alone in four cases and proved to be the most obstinate, in regard to their disappearance, of any eruptive lesion.

Palmar and plantar psoriasis appeared in thirty-five cases. Pemphigus was seen only once. The child was born with the vesicles largely developed and died shortly after.

Pustular eruptions were seen in eleven cases. Small multiple abscesses, scattered over the body, of the kind described by Vogel, containing only a granular, broken down detritus were seen in thirteen cases. These abscesses are interesting as they are apt to be overlooked, especially if accompanied by any eruption of any kind.

They are of a very low inflammatory grade, rarely giving any indication of their presence to the eye, and only to be detected on pressure. The cases in which they occurred all recovered. The mucous membranes were involved in all of my sixty-four cases. Forty-two cases had mucous patches or condylomata. Every case was affected with snuffles—nasal or naso-pharyngeal catarrh. This symptom is seldom or never absent in hereditary syphilis, and its existence in a young infant for a length of time, in spite of ordinary treatment, should be regarded with suspicion, especially if excoriations or fissures should make their appearance about the nostrils.

Diseases of the internal organs were diagnosed in twelve cases, the liver being the seat in seven cases (two of which were fatal), and in all of these latter there were present fever, jaundice, vomiting, and purging of bile, and hepatic enlargement. The lungs were affected with a deposit in three cases, presenting the usual symptoms of phthisis. In all three the deposit disappeared and the patients recovered under anti-syphilitic treatment.

No cases of enlarged spleen were diagnosed, though Dr. Gee, of London, has stated them to be present in nearly half his cases. Simple convulsions occurred in one case and continued to recur till the patient reached the age of sixteen months.

This child was supposed to be hydrocephalic, as its head was abnormally large, and the convulsions were thought to be due to this; but a suspicious nasal discharge with spots about the anus led to its being placed on anti-syphilitic treatment, and in a month's time the convulsions disappeared together with the other symptoms, and have never returned, though the patient is now over three years of age.

Convulsions with hemiplegia occurred twice, one case recovering and one dying in a second attack or relapse, at about twenty months of age, the post-mortem showing an extensive blood-clot on the surface of the brain, and a ruptured vessel. Enlargement of the thymus gland was seen only once.

Affections of the bones were seen in sixteen cases. Five of these were cases of dactylitis, bulbous enlargement of one of the phalanges of the hand or foot, first described accurately by R. W. Taylor, of New York. The others were cases of osteophytes, a variety of syphilitic bone disease which has of late been thoroughly studied and well described by Prof. M. J. Parrot, of Paris. The older writers on inherited syphilis speak of affections of the bones as extremely rare. Diday, indeed, regarding them as almost a curiosity; but the researches of the last ten or twelve years, notably by the observers just named, Taylor and Parrot, have shown them to be by no means rare.

These bony excrescences appear, according to Parrot, on the inner edge of the tibia, along the anterior surface of the femur, on the spine of the scapula, along the anterior and outer surface of the humerus and bones of the fore-arm, and occasionally on the thick and short bones of the body. It is on the cranial parietes, however, that they are most remarkable. In ten of my cases were they seen in this situation, only one presenting growths on the femur.

Parrot recognizes two varieties of bony disease in inherited syphilis —the atrophic, and, to quote his expression, osteophytic. In the first description the parts attacked take on the appearance and consistence of fruit jelly. The marrow is glistening, transparent, and in it there occurs a gradual diminution of medullary cells, which may finally even wholly disappear, and there is found only a vasculo-fibrillar mesh-work. When the hard parts are invaded they are rapidly decalcified, the spongy lamellæ literally melt away, and are replaced by large spaces, containing a tissue which has the appearance and histological structure just indicated in the altered medulla.

These changes lead to various affections, some of which may be recognized during life, notably fractures and a certain kind of weakness, termed by our authority 'syphilitic-pseudo-paralysis.'

The hairy scalp in all my cases was more or less affected, and the hair follicles of the eye-lids. This lesion gives the unpleasant aspect often noticed in syphilitic children, and in spite of treatment they seldom, even when quite large, have the luxuriant hair and large lashes of childhood. Onychia was discovered in nine cases, and was readily cured in all by a removal of the nail.

In my paper before this body, read some years since, I gave the mortality of my cases of inherited syphilis at nearly twenty-five per cent., and this rate contrasted favorably with most authorities. In my cases reported in this paper the death rate is only a fraction over fourteen per cent.; of the sixty-four children nine died.

Second and third children of syphilitic parents form quite a proportion of my cases in this communication, and of course the virulence of the disease had been modified in the parents by time, and in many cases by treatment also. But with this allowance made there is still great improvement in the death rate. Of my sixty-four cases, fourteen were children of parents who had produced, previously, syphilitic offspring. Of these none died.

The improved results I conceive to be due in the second place then, to the treatment adopted. This consists entirely in the administration of minute doses of the bichloride of mercury.

I have abandoned the inunction plan and all internal administration of any kind, except the bichloride. I give a mixture containing half a grain of the drug in three ounces of water, with a little syrup, and of this give half a teaspoonful every hour or two to begin with. By this treatment we avoid the dirtiness and greasiness and trouble of the inunction plan—and the time and trouble are no small items with laboring people—and we also shun the disagreeable effects often following the administration of small doses of calomel. Since my first adoption of the bichloride treatment, over four years ago, I have never seen a bad result from its use. My plan is to give it until all traces of the disease have disappeared, and then to give it in "tonic" doses for a month or six weeks longer. The dose is a ninety-sixth of a grain, and giving it in "tonic" doses is only giving it thrice daily, instead of every hour, as at first.

